# Defining Prolonged Length of Acute Care Stay for Surgically and Conservatively Treated Patients with Spontaneous Intracerebral Hemorrhage: A Population-Based Analysis

**DOI:** 10.1155/2016/9095263

**Published:** 2016-03-27

**Authors:** Marco Stein, Björn Misselwitz, Gerhard F. Hamann, Malgorzata A. Kolodziej, Marcus H. T. Reinges, Eberhard Uhl

**Affiliations:** ^1^Department of Neurosurgery, Justus-Liebig University Giessen, Klinikstrasse 33, 35392 Giessen, Germany; ^2^Institute of Quality Assurance Hesse, Frankfurter Strasse 10-14, 65760 Eschborn, Germany; ^3^Department of Neurology and Neurological Rehabilitation, Bezirkskrankenhaus Günzburg, Ludwig-Heilmeyer-Strasse 2, 89312 Günzburg, Germany

## Abstract

*Background*. The definition of prolonged length of stay (LOS) during acute care remains unclear among surgically and conservatively treated patients with intracerebral hemorrhage (ICH).* Methods*. Using a population-based quality assessment registry, we calculated change points in LOS for surgically and conservatively treated patients with ICH. The influence of comorbidities, baseline characteristics at admission, and in-hospital complications on prolonged LOS was evaluated in a multivariate model.* Results*. Overall, 13272 patients with ICH were included in the analysis. Surgical therapy of the hematoma was documented in 1405 (10.6%) patients. Change points for LOS were 22 days (CI: 8, 22; CL 98%) for surgically treated patients and 16 days (CI: 16, 16; CL: 99%) for conservatively treated patients. Ventilation therapy was related to prolonged LOS in surgically (OR: 2.2, 95% CI: 1.5–3.1; *P* < 0.001) and conservatively treated patients (OR: 2.5, 95% CI: 2.2–2.9; *P* < 0.001). Two or more in-hospital complications in surgical patients (OR: 2.7, 95% CI: 2.1–3.5) and ≥1 in conservative patients (OR: 3.0, 95% CI: 2.7–3.3) were predictors of prolonged LOS.* Conclusion*. The definition of prolonged LOS after ICH could be useful for several aspects of quality management and research. Preventing in-hospital complications could decrease the number of patients with prolonged LOS.

## 1. Introduction

Between 10 and 20% of all strokes are caused by spontaneous intracerebral hemorrhage (ICH) [1]. Spontaneous ICH is associated with significant morbidity and mortality worldwide. Length of hospital stay (LOS) is a factor directly related to hospital costs and is influenced by different variables [2]. In-hospital complications after stroke were known to be associated with longer LOS [3]. However, most available stroke data about in-hospital complications concerns ischemic stroke and few publications focus on ICH [4, 5]. Data about prolonged LOS after ICH and influencing factors were only available for a small Asian cohort [6]. Currently, no population-based data about the definition of prolonged LOS with a focus on ICH exists in Europe. Furthermore, in the available publications on this topic, no difference was made between surgically and conservatively treated patients with ICH [7]. In this context, the differentiation between ICH patients with and without ventilation therapy is important and insufficiently depicted in the available publications. However, data about this topic are needed for the improvement of quality assessment, for future research, and, last but not least, for the development of adequate and realistic remuneration systems for patients with ICH.

Data for LOS are shown in timelines highlighting potential change points characterized by changes in data distribution or data spread. These change points are often essential for data interpretation. Change-point analysis also has the power to detect and determine these change points retrospectively, as well as to test them for statistical significance. Today, change-point analyses are widely used in engineering, economics, and natural sciences [8–10].

The purpose of this study was to evaluate LOS among surgically and conservatively treated patients with spontaneous ICH during acute care. We set out to investigate various aspects associated with LOS that include comorbidities, in-hospital complications, ventilation therapy, and patients' baseline characteristics. Using change-point analysis, we aimed to calculate different change points for a prolonged LOS for surgically and conservatively treated patients.

## 2. Methods

The dataset for this study was obtained from a large prospective stroke database in the state of Hesse, Germany. The Hessian Stroke Registry is mandatory for all stroke patients diagnosed with ischemic stroke (ICD-10: I63), ICH (ICD-10: I61), and subarachnoid hemorrhage (ICD-10: I60). For this study we only included patients diagnosed with spontaneous ICH (ICD-10: I61.0 to I61.9), with an admission date between January 1, 2007, and December 31, 2014.

The term “pre-ICH disability” was defined as a modified Rankin Scale (mRS) of 3–5 prior to ICH onset. Initial Glasgow Coma Score (GCS) was assessed at hospital admission. In-hospital complications were recorded during the acute care stay and were categorized in 1, 2, and ≥3 secondary complications.

The basis for the data registration in the Hessian stroke database was regulated by the German Social Code, Book Five. Due to the anonymous data collection for this study, neither institutional board approval nor informed consent was required.

### 2.1. Statistical Methods

For the comparison of categorical variables, the chi-square test was used. Continuous variables were analysed by the Student *t*-test or the Mann-Whitney *U* Test. We analysed the relation between pre-ICH existing comorbidities, baseline characteristics, and in-hospital complications to a prolonged LOS for surgically and conservatively treated patients in a multivariate regression model. Variables with a *P* value < 0.1 in a univariate analysis were included in the multivariate analysis. Odds ratios (OR) and 95% confidence intervals (CI) were calculated to observe the relation between the quantity of in-hospital complications and the chance of a prolonged LOS for surgically and conservatively treated patients. These analyses were performed with SPSS 21 for Windows (IBM Inc., Armonk, NY, USA). Cut-off values for the duration of ventilation therapy were calculated by receiver operating characteristic (ROC) analysis with MedCal Vers.15.8 for Windows (MedCalc Software, Ostend, Belgium). Youden's index (sensibility + specificity − 1) and the area under the curve (AUC) were calculated. The highest available Youden's index was defined as the best cut-off point for the duration of prolonged LOS in surgically and conservatively treated patients.

### 2.2. Change-Point Analysis

The cumulative sums (CUSUM) were calculated by the method of Taylor [11]. Sudden turns in the CUSUM curve direction represent changes in data average. A CI for every change point was calculated. The closer a CI is, the more accurate the time of the change point can be pinpointed. The confidence level (CL) shows how frequently the calculated change point will likely occur. Further information provided by change-point analysis is the level of the calculated change point. A level of 1 indicates a high importance of the change. In our analysis, only levels 1 and 2 changes were accepted to be included. If outliers in the LOS data timeline led to a violation of independent error assumption, the group rows of the change-point analysis were compared to groups of two rows.

## 3. Results

### 3.1. Basic Characteristics

Overall, 13292 patients with the diagnosis of spontaneous ICH were identified in the study period. In 20 patients (0.2%), LOS data were missing. The remaining 13272 patients were included in the analysis. Median age of the patients was 67 years (range: 19–93 years). Approximately half of the cohort, 6376 patients (48.0%), was female. Median GCS was 12 (IQR: 7–15). Secondary expansion of the ICH into the ventricles was observed in 1777 patients (13.4%). Preexisting comorbidities like arterial hypertension, diabetes, hypercholesterolemia, atrial fibrillation, prior insult, and other comorbidities were observed in 9799 (73.8%), 2045 (15.4%), 1687 (12.7%), 2426 (18.3%), 2039 (15.4%), and 3199 (24.1%) of the patients, respectively. A pre-ICH disability was documented in 2062 patients (15.4%). During the acute care stay, operations of the hematoma were performed in 1405 (10.6%) patients. Ventilation therapy in the intensive care unit was documented in 3438 (25.9%) patients. The median ventilation time was four days (IQR: 1–13). In-hospital complications that occurred with an incidence >1% are listed in Table 1. Overall, 37.1% of patients (*N* = 4927) had one or more complications during acute care stay. The total in-hospital mortality rate was 23.6% (3138 patients), and the median mRS of the survivors at hospital discharge was four with an IQR of two to five.

### 3.2. The Calculation of Prolonged Intensive Care Stay

**Figure 1:**
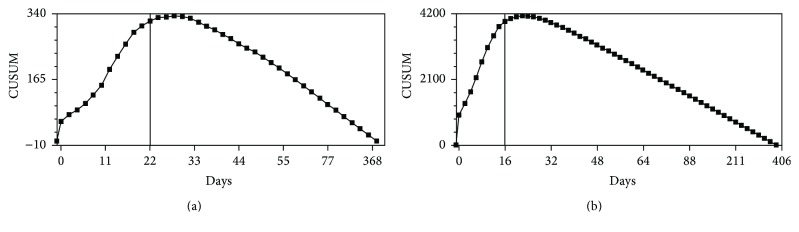
Timelines of cumulative sum control (CUSUM) analysis. CUSUM analysis by days of acute care stay for surgically (a) and conservatively treated (b) patients. The calculated change points were 22 days for surgically treated patients and 16 days for conservatively treated patients with spontaneous intracerebral hemorrhage.

The calculated change points for a prolonged LOS at acute care were 22 days (CI: 8, 22; CL 98%) for surgically treated patients and 16 days (CI: 16, 16; CL: 99%) for conservatively treated patients. The CUSUM charts by days of acute care stay are shown in Figures 1(a) and 1(b). A subgroup analysis by ventilation therapy for surgically and conservatively treated patients is listed in Table 2.

### 3.3. Comparing Surgical and Conservative Patients

The rate of patients with ventilation therapy in the ICU was increased in surgically treated patients compared to conservatively treated patients (1060 patients (75.4%) versus 2378 patients (20.0); *P* < 0.001). Median acute care stay after ICH was significantly longer in surgically treated patients compared to conservatively treated patients (15 days, IQR: 8–23, versus 9 days, IQR: 3–14; *P* < 0.001). By defining prolonged LOS as >22 days for surgically treated patients and >16 days for conservatively treated patients, 366 (26.0%) patients of the surgical group and 2289 (19.3%) patients of the conservative group with prolonged LOS were identified. Differences in prior ICH comorbidities, baseline characteristics, and in-hospital complications for prolonged and nonprolonged LOS for both groups are shown in Table 3.

Ventilation therapy was associated with prolonged LOS in a univariate analysis for surgically and conservatively treated patients. Not only did the quantity of patients with ventilation therapy increase, but the duration of ventilation therapy was also longer in patients with prolonged LOS when compared to nonprolonged LOS.

In an ROC analysis of ventilation therapy for surgically treated patients, we observed an LOS of >9 days (Youden's index: 0.646; AUC: 0.889, 95% CI: 0.875–0.901; *P* < 0.001) as the optimal cut-off point to predict prolonged LOS. For conservatively treated patients, the optimal cut-off point of ventilation therapy was detected at >17 days (Youden's index: 0.489; AUC: 0.821, 95% CI: 0.796–0.843; *P* < 0.001).

### 3.4. Binary Regression Analysis

The main predictors of prolonged LOS for surgically and conservatively treated patients are shown in Table 4. In the surgical group, the presence of two or more in-hospital complications and ventilation therapy were identified as independent predictors of prolonged LOS. The observed age and GCS were lower in surgically treated patients with prolonged LOS compared to patients without prolonged LOS. GCS was no longer a significant predictor after adjustment for age, ventilation therapy, and ≥2 in-hospital complications. In the conservative group, at least one in-hospital complication and ventilation therapy were independently associated with prolonged LOS. Patients with a prolonged LOS were younger compared to patients without prolonged LOS. The observed GCS was slightly higher in patients with a prolonged LOS (Table 3) compared to patients with nonprolonged LOS.

## 4. Discussion

### 4.1. Summary of Findings

With the definition of prolonged LOS as >22 days for surgically treated patients and >16 days for conservatively treated patients after spontaneous ICH, every fourth patient in the surgical, and approximately every fifth patient in the conservative group, shows a prolonged LOS during acute care in Central Germany. No representative statement of prolonged or nonprolonged LOS can be made when only observing the median or mean LOS. To the best of our knowledge, this is the first definition of prolonged LOS for surgically and conservatively treated patients after spontaneous ICH. Furthermore, change points for prolonged LOS were higher among surgically treated and ventilated patients (22 days; CI: 18–22), compared to conservatively treated patients with ventilation therapy (10 days; CI: 6–10). Moreover, the calculated change points for LOS of nonventilated patients were higher in surgically treated patients (19 days; CI: 15–21) when compared to conservatively treated patients (>16 days; CI 10–16). In this context, the majority of patients in the surgical group were ventilated and the majority of conservatively treated patients were nonventilated. This explains that the same change points for these groups with different CI and CL were detected.

Publications on the topic of prolonged LOS are rare, especially for patients with ICH [6]. Most publications that evaluate prolonged LOS after stroke exclusively depict ischemic stroke [12] or define prolonged acute care stay with a scoring system without differentiation for ischemic stroke or intracerebral hemorrhage or for patients with and without ventilation therapy [13]. The association between comorbidities and LOS for stroke patients was controversial in several studies [13–16]. In our data, the presence of arterial hypertension was only associated with prolonged LOS for conservatively treated patients. Stroke severity is a predictor of prolonged LOS in stroke patients [13, 14]. However, these publications are not specifically focused on patients with ICH and represent mixed cohorts of ischemic stroke and ICH, and no differentiations in the calculation of prolonged LOS between surgical and conservative treatment were made.

Severity of ICH was documented with the GCS at admission in our data, and a lower GCS was associated with prolonged LOS of surgically treated patients in univariate analysis. After adjustment for several predictors in a binary logistic regression model (Table 4), GCS no longer remains a predictor of prolonged LOS. In-hospital complications are a known predictor of LOS in stroke patients [3] and in patients with ICH [17]. In a previous publication of our study group, two or more in-hospital complications in surgically treated patients and at least one or more in-hospital complications in conservatively treated patients were associated with higher rates of in-hospital mortality [5]. In the current analysis on prolonged LOS, the accumulation of two or more in-hospital complications in the surgically treated group and the presence of at least one or more in-hospital complications in the conservative group were related to higher chance of prolonged LOS during acute care stay. Ventilation therapy was also a predictor of prolonged LOS [18]. In line with previous studies, ventilation therapy was an important and unsurprising predictor of prolonged LOS, independent of the treatment form in our data. Furthermore, we were able to calculate significant cut-off values for the duration of ventilation therapy by ROC analysis.

### 4.2. Strengths and Limitations

The strengths of our study are the large cohort of patients, the availability of prospectively collected detailed data, the method of change-point analysis, the analysis of prolonged LOS for several conditions (surgical and conservative treatment, both with and without ventilation therapy), and the population-based design. However, some limitations of our study should be discussed. Our study was not a randomized trial for surgical treatment of intracerebral hematomas, and the indication for surgery was not standardized. In the used database, hematoma volumes of the patients were not available. Hematoma volume is a known predictor of mortality and could influence the course of acute care stay. However, several publications observed a similar predictive value of the GCS at admission alone compared to scoring systems that include hematoma volume [19–21]. Furthermore, hematoma volume was not a predictor of prolonged LOS in a previous study [6].

Patients with ICH may experience several medical complications during acute care stay; in the used database not all possible complications are listed in detail (e.g., hyperglycemia). However, several known important predictors and variables during acute care stay were analysed in our data such as comorbidities, age, gender, GCS, the presence of secondary IVH, duration of ventilation therapy, in-hospital complications, in-hospital mortality, and LOS.

## 5. Conclusion

For the first time, change points for LOS during acute care were defined for surgically and conservatively treated patients with ICH. Furthermore, we could identify different change points for prolonged LOS for surgically and conservatively treated patients, both with and without ventilation therapy after spontaneous ICH. The calculated values for the definition of prolonged acute care stay could be useful for several aspects of quality management, research, and planning negotiations with health insurance groups in order to develop adequate and realistic remuneration systems for the acute care therapy of patients with spontaneous ICH.

## Figures and Tables

**Table 1:** In-hospital complications for patients with spontaneous intracerebral hemorrhage.

*N* (%)	13272 (100.0)
Pulmonary	1981 (14.9)
Brain edema	823 (6.2)
Urogenital	767 (5.8)
Cardial	756 (5.7)
Hydrocephalus	554 (4.2)
Epilepsy	488 (3.7)
Rebleeding	453 (3.4)
Sepsis	231 (1.7)
Brain infarction	178 (1.3)
Other	1661 (12.5)

(Only secondary complications with an occurrence >1% are listed.)

**Table 2:** Results of a change-point analysis for prolonged length of stay (LOS). Median LOS and calculated change points in LOS timeline by surgical and conservative treatment, both with and without ventilation therapy.

Patients	Days (Median)	Days (IQR)^*∗*^	Prolonged LOS (Change point, days)	Confidence interval	Confidence level
Conservative (*N* = 11867)	9	3–14	16	16, 16	99%

Surgical (*N* = 1405)	15	8–23	22	8, 22	98%

Ventilation Conservative (*N* = 2378)	6	1–20	10	6, 10	98%

Ventilation Surgical (*N* = 1060)	16	9–25	22	18, 22	99%

Nonventilated Conservative (*N* = 9489)	9	4–14	16	10, 16	95%

Nonventilated Surgical (*N* = 345)	12	2–17	19	15, 21	100%

^*∗*^Interquartile range (P25–P75).

**Table 3:** Main characteristics of 13272 patients with spontaneous intracerebral hemorrhage (ICH). Baseline characteristics are presented by prolonged and nonprolonged length of stay (LOS) for surgical and conservative treatment.

*N* (%)	Surgical group			Conservative group		
	Nonprolonged LOS (≤22 days) *N* = 1039	Prolonged LOS (>22 days) *N* = 366	*P* value	Nonprolonged LOS (≤16 days) *N* = 9578	Prolonged LOS (>16 days) *N* = 2289	*P* value
Female	469 (45.1)	154 (42.1)	0.310	4726 (49.3)	1027 (44.9)	<0.001
Age^*∗*^	68 (56–75)	66 (52–74)	0.006	76 (67–83)	73 (63–80)	<0.001
Pre-ICH disability	81 (7.8)	34 (9.3)	0.199	1594 (16.6)	353 (15.4)	0.170
GCS at admission^*∗*^	10 (3–14)	7 (3–13)	0.001	13 (7–15)	13 (8–15)	0.043
Secondary IVH	113 (10.9)	40 (10.9)	0.978	1288 (13.4)	336 (14.7)	0.124
Ventilation therapy	739 (71.1)	321 (87.5)	<0.001	1640 (17.1)	738 (32.2)	<0.001
Duration ventilation therapy, days^*∗*^	5 (2–11)	20 (9–28)	<0.001	2 (1–4)	17 (8–24)	<0.001
Comorbidities:						
Arterial hypertension	651 (62.7)	232 (63.4)	0.803	7134 (74.5)	1782 (77.9)	0.001
Diabetes mellitus	114 (11.0)	43 (11.7)	0.685	1507 (15.7)	381 (16.6)	0.284
Hypercholesterolemia	92 (8.9)	29 (7.9)	0.585	1244 (13.0)	322 (14.1)	0.171
Atrial fibrillation	182 (17.5)	65 (17.8)	0.916	1771 (18.5)	408 (17.8)	0.460
Prior insult	96 (9.2)	32 (8.7)	0.777	1559 (16.3)	352 (15.4)	0.293
Other	303 (29.2)	118 (32.2)	0.269	2202 (23.0)	576 (25.2)	0.027
In-hospital complications:						
1 complication	318 (30.6)	131 (35.8)	0.067	2130 (22.2)	724 (31.6)	<0.001
2 complications	145 (14.0)	101 (27.6)	<0.001	539 (5.6)	379 (16.6)	<0.001
≥3 complications	71 (6.8)	64 (17.5)	<0.001	143 (1.5)	182 (8.0)	<0.001
In-hospital mortality	167 (16.1)	20 (5.5)	<0.001	2775 (29.0)	176 (7.7)	<0.001
Length of hospital stay, days^*∗*^	12 (5–16)	31 (26–43)	<0.001	7 (2–11)	23 (19–29)	<0.001

^*∗*^Median and IQR (P25–P75).

**Table 4:** Predictors of prolonged length of stay (LOS) during acute care. Results for the prediction of prolonged LOS in a binary logistic regression model are shown by surgical and conservative treatment.

Predictor	OR (95% CI)	Adjusted OR (95% CI)	*P *value
Surgical group (*N* = 1405)			
Age	0.988 (0.980–0.996)	0.988 (0.980–0.997)	0.011
GCS	0.958 (0.933–0.983)	0.988 (0.961–1.016)	0.384
Ventilation therapy	2.896 (2.062–4.067)	2.176 (1.511–3.136)	<0.001
≥2 in-hospital complications	3.128 (2.425–4.035)	2.701 (2.077–3.512)	<0.001

Conservative group (*N* = 11867)			
Age	0.987 (0.983–0.990)	0.988 (0.985–0.992)	<0.001
GCS	1.011 (1.000–1.021)	1.078 (1.063–1.092)	<0.001
Ventilation therapy	2.303 (2.079–2.552)	2.505 (2.193–2.861)	<0.001
≥1 in-hospital complications	3.080 (2.805–3.381)	2.986 (2.701–3.300)	<0.001
Arterial hypertension	1.204 (1.080–1.343)	1.231 (1.094–1.386)	0.001
Other comorbidities	1.126 (1.013–1.252)	1.023 (0.915–1.145)	0.687

## Abbreviations

AUC: Area under the curve
CI: Confidence interval
CL: Confidence level
CUSUM: Cumulative sum
GCS: Glasgow Coma Score
ICH: Intracerebral hemorrhage
IQR: Interquartile range
IVH: Intraventricular hemorrhage
LOS: Length of stay
OR: Odds ratio
ROC: Receiver operating curve
SD: Standard deviation.
